# Faucet aerators as a reservoir for Carbapenem-resistant *Acinetobacter baumannii*: a healthcare-associated infection outbreak in a neurosurgical intensive care unit

**DOI:** 10.1186/s13756-019-0635-y

**Published:** 2019-12-30

**Authors:** Yu Lv, Qian Xiang, Ying Z. Jin, Ying Fang, Yu J. Wu, Bin Zeng, Hua Yu, Hong M. Cai, Qiong D. Wei, Chen Wang, Jing Chen, Hui Wang

**Affiliations:** 1 Healthcare-associated Infections Control Center, Sichuan Academy of Medical Sciences and Sichuan People’s Hospital, Chengdu, Sichuan People’s Republic of China; 2 Healthcare-associated Infections Control Center, Affiliated Chinese Medicine Hospital of Southwestern Medical University, LuZhou, Sichuan People’s Republic of China; 3 Department of Nursing, Jianyang People’s Hospital, Jianyang, Sichuan People’s Republic of China; 4 Neurosurgical Intensive Care Unit, Sichuan Academy of Medical Sciences and Sichuan People’s Hospital, Chengdu, Sichuan People’s Republic of China; 5 Microbiology laboratory, Sichuan Academy of Medical Sciences and Sichuan People’s Hospital, Chengdu, Sichuan People’s Republic of China

**Keywords:** Healthcare-associated infection, Outbreak, *Acinetobacter baumannii*, Emergency response, Neurosurgical intensive care unit, Faucet aerator.

## Abstract

**Background:**

On January 7, 2019, we observed an outbreak of healthcare-associated infection (HAI) caused by Carbapenem-resistant *Acinetobacter baumannii* (CRAB) in the neurosurgical intensive care unit (NSICU). A follow-up epidemiological investigation was conducted, and an emergency response was initiated. We aimed to study the clonal transmission of CRAB and its possible source.

**Methods:**

A matched case-control (1:2) study was performed to identify the possible predisposing factors. A multifaceted intervention was implemented to control the outbreak. We collected environmental samples from patients’ rooms and living area of the staff. CRAB isolates were tested for genetic relatedness by Pulsed-Field Gel Electrophoresis (PFGE).

**Results:**

Environmental sampling showed that a faucet aerator was contaminated with *A. baumannii*. Molecular typing revealed the only outbreak strain, which was isolated from tracheal aspirate cultures of the first case of community-acquired infection and 3 cases of HAI. In environmental samples, the outbreak strain was found only in the faucet aerator of the dining room. This CRAB outbreak was discovered in time, and further progress of this outbreak was prevented through a pre-set emergency response procedure.

**Conclusions:**

The faucet aerator acted as a reservoir for bacteria in the outbreak, and contamination of the faucet aerator might have occurred from splashes originating from handwashing by the healthcare workers (HCWs). In high-risk areas, such as NSICU, the faucet aerators should not be used during an outbreak or they should be regularly cleaned and disinfected. The start-up criteria for the emergency response played a key role in controlling the CRAB outbreak, and its settings should be discussed more widely.

## Introduction

Carbapenem-resistant *Acinetobacter baumannii* (CRAB) is emerging as a problematic pathogen for patients, clinicians, and infection-control personnel, owing to high mortality, less treatment options, and its ability to contaminate and persist in the healthcare environment at high levels [1]. In the first ever list of the deadliest superbugs that threaten human health published by the World Health Organization (WHO) in 2017, CRAB was listed in the “critical” section [2]. According to data from the China Antimicrobial Surveillance Network (CHINET), the detection rate of CRAB has increased rapidly in the last 10 years. By 2017, about 66.7 and 69.3% of *A. baumannii* strains were resistant to imipenem and meropenem [3]. A multi-center study in China showed that the incidence density of all CRAB isolates was 2.47 per 1000 inpatient-days in the intensive care unit (ICU), which was significantly higher than that previously reported in other regions [4, 5]. The prevalence of CRAB in China is at high levels, and CRAB has been increasingly reported as a cause of nosocomial outbreaks in ICUs [6].

A lot of epidemiological and molecular evidence suggests that there is a close correlation between healthcare-associated infection (HAI) outbreaks caused by nonfermentative gram-negative bacilli (NFGNB) and contaminated tap water in ICUs [7–9]. Legionella, *S. maltophilia* and *Pseudomonas aeruginosa* are the most commonly found pathogens in these studies, and relatively few cases of HAI outbreaks caused by *A. baumannii* (AB) have been reported.

The faucet aerator played a key role in the tap water pollution process in previous outbreak cases [10, 11]. In an outbreak investigation in Taiwan, Wang found that one-third of the ICU faucet aerators sampled were contaminated with NFGNB [12]. Verweij inferred that the contaminated aerator screens of tap water outlets were the source of contamination after an investigation of the outbreak caused by *S. maltophilia* [13].

**Fig. 1:**
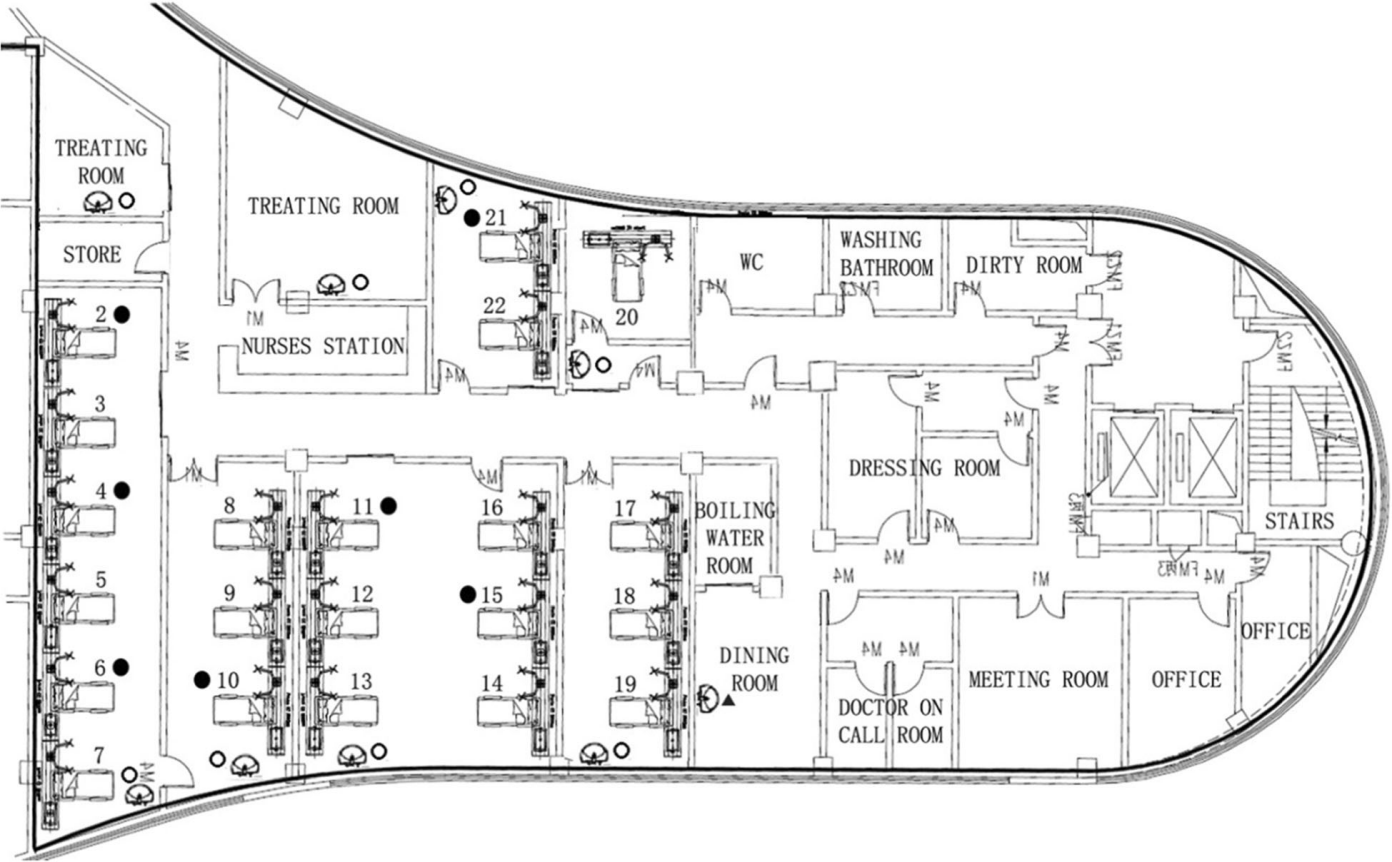
Schematic map of NSICU. The symbol in the figure indicates the locations of samples that were positive for CRAB. (●) Patients with isolates positive for CRAB. (▲) Environmental samples positive for CRAB. (〇) Environmental samples from sinks negative for CRAB

From December 2018 to January 2019, 7 patients had CRAB positive cultures isolated from the lower respiratory tract in the neurosurgical ICU (NSICU) of the largest tertiary A-level hospital in Sichuan Province, China. All clinical isolates were resistant to carbapenems, and their antibiotic-susceptibility patterns were identical. Three of these 7 patients developed lower respiratory tract infection due to CRAB, and they had similar symptoms and signs. This NSICU was remodeled from a previous rehabilitation ward, and it consisted of two 6-bedded rooms, two 3-bedded rooms, one 2-bedded room, and one isolation room. A map of the NSICU is shown in Fig. 1. Herein, we describe a follow-up epidemiological investigation that included environmental sampling and genotyping by Pulsed-Field Gel Electrophoresis (PFGE).

## Materials and methods

### Epidemiological investigation

The tools provided in the “Guideline for control of an healthcare-associated infection outbreak (WS/T 524-2016)” were used for epidemiological investigation [14]. An outbreak emergency start-up procedure was implemented to detect and contain the HAI outbreak.

Emergency response criteria for a HAI outbreak were as follows:

Since the detection rate of a certain pathogenic microorganism in clinical specimens from a certain department has significantly increased, all the past cases within 7 calendar days of the department are reviewed from the date of specimen submission, and the emergency response is initiated if the following conditions are met:

- ① Within 7 calendar days, there were 2 cases or more of HAI or suspected cases of HAI caused by the same pathogenic microorganisms with highly similar antibiotic-susceptibility patterns;
- ② Within 3 calendar days, there were 3 cases or more of hospital-acquired cases (including colonization, HAI, and suspected HAI) caused by the same pathogenic microorganisms with highly similar antibiotic-susceptibility patterns;

With reference to the definition of Repeat Infection Timeframe (RIT) for HAI surveillance by the US National Healthcare Safety Network (NHSN) [15], we determined the following exclusion criteria:

- ① Cases that were judged to be contaminated by the pathogenic microorganisms or were taken outside the department;
- ② Patients whose infection had been cured;
- ③ Patients who had been hospitalized for a long time (excluding patients who had been hospitalized for more than 14 days after infection, but did not include new infections that have been identified), thereby avoiding frequent triggering of an emergency response by these patients.

The outbreak period was defined from December 28, 2018 to January 19, 2019. HAIs were defined according to the “HAI Diagnostic Criteria” issued by the Ministry of Health of the People’s Republic of China in 2001 [16].

### Hand hygiene compliance observation

Since January 2016, the HAI management department and the nursing department of our hospital have jointly established a hand hygiene compliance monitoring team. After unified training, the team members used the WHO hand hygiene tools to measure monthly hand hygiene compliance in 92 wards of the hospital. To avoid observation bias, the observation unit was temporarily randomly assigned to each team member. During the outbreak, daily hand hygiene compliance supervision was performed by the NSICU head nurse using the same method.

### Case-control study

A matched case-control study was performed to verify the suspicious factors leading to the HAI outbreak. The cases of HAI were defined as those who had stayed in the ICU for at least 48 h with CRAB infection or colonization, but it did not include cases of CRAB contamination and community infections.

Controls were patients who met the matching criteria. For each case patient, 2 control patients were randomly selected from the group of patients admitted to the NSICU during the same period who did not acquire CRAB.

Matching criteria were as follows:

- ① Patients with the same gender and in the same age group as the cases, and the age difference should be within 5 years;
- ② To avoid “time bias,” the selected controls should have sufficient length of hospitalization stay [17, 18]. Therefore, controls were patients who had been in the NSICU for an interval at least as long as that between the time of NSICU admission and isolation of CRAB for the case patient;
- ③ The Glasgow Coma Scale (GCS) of the controls should be in the same category as the cases, and the GCS difference should be within 2 scales. The GCS has a well-established profile for use in people who have sustained a traumatic brain injury, designating them into three severity categories; mild (GCS 13–15), moderate (9–12), and severe (3–8) categories [19].

### Study sample

Both clinical and environmental specimens were processed using standard techniques and reagents. Environmental sampling was performed after cleaning the entire unit. Environmental specimens were collected from suspected potential pollutants, including bed rails, door handles, curtains, computer keyboards and mice, ventilator control panel and sensor, air vents, mattresses, treatment carts, equipment tower surfaces, used rag and towel, staff hands, used medical textiles, sink inner surface, potable water, aerators, the outer surface of the faucets, and the inner surface of the proximal end of the water outlet. The entire exterior of aerators was sampled after dismantling. Pre-moistened cotton swabs were used to sample environmental specimens. The swabs were immediately inoculated onto sheep blood agar plates and incubated at 37 °C for 2 to 4 days.

### Microbiological methods

The standard paper diffusion method and Vitek-2 (BioMerieux, France) automatic instrument detection was used to detect the sensitivity of AB to 21 commonly used antibiotics, including imipenem, meropenem, doripenem, ticarcillin, ampicillin/sulbactam, ceftriaxone, cefotaxime, cefepime, cefotetan, cefuroxime, cefoperazone/sulbactam, piperacillin/sulbactam, gentamicin, tobramycin, ciprofloxacin, amikacin, tetracycline, tigecycline, minocycline, amoxicillin/clavulanic acid, and aztreonam. Molecular typing was performed by the CHEF-Mapper PFGE system, and PFGE pattern clustering analysis was conducted by using BioNumerics Version 6.64. The PFGE classification was judged according to the discriminant proposed by Tenover et al. [20]. Differences of more than 3 bands were considered to be of different types.

### Infection control interventions

Intensive infection control measures were implemented according to the guideline (WS/T 524–2016) during the early outbreak (January 8–12, 2019). They included the following: (1) Strengthening measures to improve hand hygiene compliance were implemented. Healthcare workers’ (HCWs) hand hygiene compliance was checked twice daily, and violators were financially penalized. (2) Isolation was strictly enforced. Colonized/infected patients were separated into concentrated areas. (3) Fluorescent labeling was used to control the daily cleaning and disinfection effect of the ICU environment surface. (4) Aerosolized hydrogen peroxide was used to carry out terminal disinfection of individual wards in turn. (5) Unnecessary transfer of patients from other units or surrounding hospitals was stopped. (6) Contact precautions were practiced for all patients. (7) Medical staff and cleaning staff were retrained for an emergency response to the HAI outbreak.

From January 13, 2019, the use of all faucet aerators in the NSICU was prohibited. To avoid the infection risk of water-borne bacterial contamination due to splashing after the outbreak, all aerators were immersed once a week with chlorine disinfectant.

### Statistical methods

Statistical analysis of the data was performed using SPSS 23.0 software. A conditional logistic regression model for matched case-control groups was used to identify the factors associated with CRAB colonization and infection. All tests were 2-sided with an level of 0.05.

## Results

### Outbreak investigation and response

**Fig. 2:**
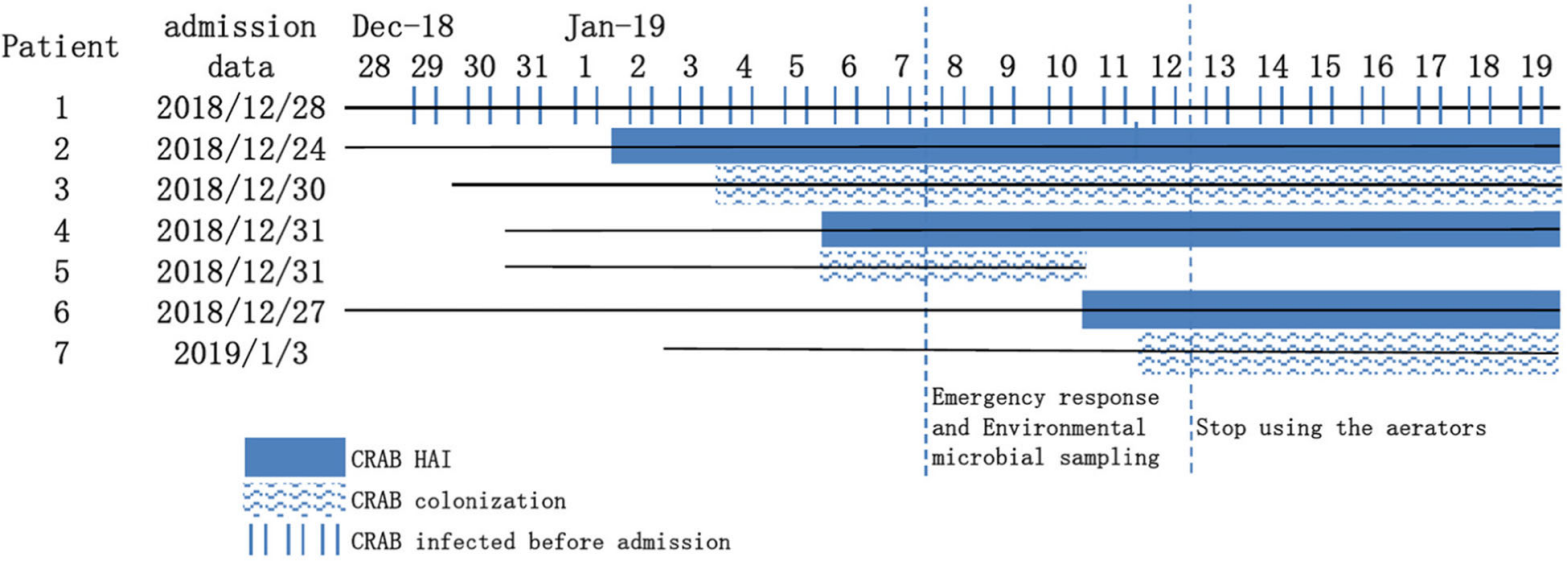
Progress of the CRAB outbreak in the NSICU. The solid line indicates the patients’ hospitalization status in the NSICU. The blue solid bar indicates that the patient is in CRAB HAI status. The wavy texture bar indicates that the patient is in CRAB colonization status. The Vertical bar represents the patient who was infected with CRAB before NSICU admission

On January 7, 2019, the Infection Control Department was notified that the tracheal aspirate culture results of 5 patients in the NSICU showed CRAB positivity. Of these 5 patients, 1 had CRAB infection before admission, 2 had CRAB HAI, and 2 had CRAB colonization. This state satisfied our pre-established emergency response criteria for HAI outbreaks, and therefore, following the emergency response process, an outbreak control team was established including an infection control officer, bacteriologists, cleaning staff, NSICU doctors, and nurses. Intensive infection control measures and environmental microbial sampling were implemented immediately, but their effects were poor. Three days after the emergency response, a new case of infection and a case of colonization occurred. However, after the use of all faucet aerators in the NSICU was prohibited on January 13, 2019, there were no new cases of infection or colonization, other than repeated detection in previous patients, until March 24, 2019, when CRAB was identified in a patient’s airway aspirate specimen. The timeline of the outbreak investigation is illustrated in Fig. 2.

### Isolation of CRAB

**Fig. 3:**
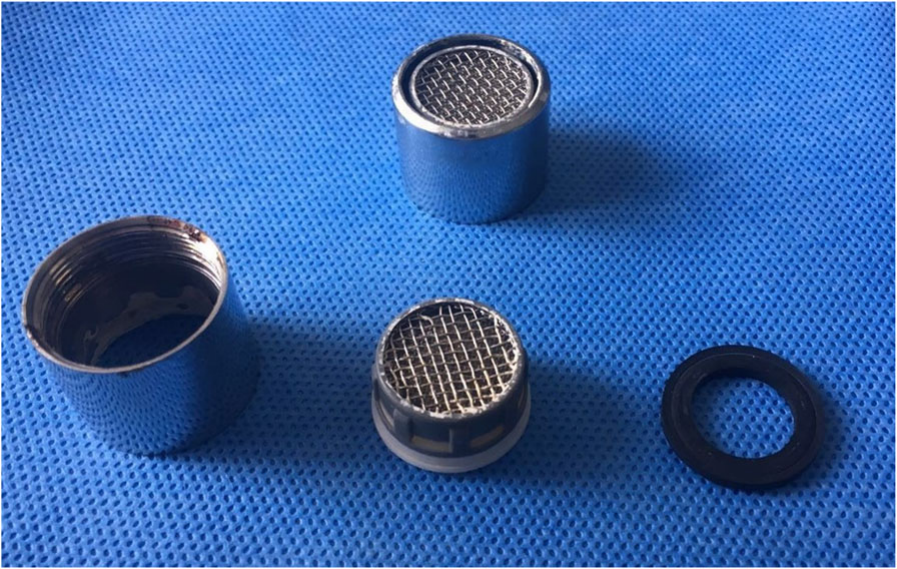
Photograph of the faucet aerator. The faucet aerators are made of several wire mesh to filter impurities in water and prevent splashing

All lower respiratory tract samples were obtained from tracheal aspirates. Within 3 days from the initiation of the emergency response on January 7, 2019, 200 environmental samples were collected. Only one sample, which was obtained from the faucet aerator in the dining room, was found to be culture positive. The photograph of the faucet aerator is shown in Fig. 3. All detected AB were only sensitive to tigecycline and resistant to other antibiotics.

### Case–control study

Of the 6 cases, 3 scored 4 points on the GCS, 2 scored 5 points on the GCS, and 1 scored 9 points on the GCS. The mean age (±SD) of cases was 70.50 ± 7.31 years. Six (50%) were men. Three patients (50%) had lower respiratory tract infection due to CRAB, and the remaining 3 patients had only CRAB colonization of the lower respiratory tract. A comparison of the possible risk factors for CRAB acquisition in cases and controls is shown in Table 1. On univariate analysis or by using the multivariate model, cases and controls did not differ significantly with respect to the studied characteristics.

**Table 1:** Results of unadjusted conditional logistic regression modeling of the possible risk factors for CRAB acquisition by cases and controls in the neurosurgical intensive care unit (NSICU)

Possible risk factor	Case patients (*n* = 6)	Control patients (*N* = 12)	Univariate OR (95% CI)^a^	*P*
Blood transfusion or the use of blood products	2 (33.33%)	5 (41.67%)	0.70 (0.09–5.43)	1.000
Urinary catheterization	6 (100.00%)	12 (100.00%)	…	…
Hemodialysis	0 (0.00%)	0 (0.00%)	…	…
Venous catheterization	6 (100.00%)	6 (50.00%)	…	0.054
Mechanical ventilation	5 (83.33%)	8 (66.67%)	2.50 (0.21–29.26)	0.615
Tracheotomy	5 (83.33%)	6 (50.00%)	5.00 (0.44–56.62)	0.316
Hypertension ^b^	5 (83.33%)	5 (41.67%)	7.00 (0.61–79.87)	0.152
Diabetes	0 (0.0%)	1 (8.33%)	…	1.000
Chronic obstructive pulmonary disease	1 (16.67%)	0 (0.00%)	…	0.333
Tumor	0 (0.00%)	0 (0.00%)	…	…
Surgery	4 (66.67%)	4 (33.33%)	0.25 (0.03–2.00)	0.191
Medical group	3 (50.00%)	7 (58.33%)	0.71 (0.10–5.12)	0.738

NOTE. Data are no. (%) of objects. CI: confidence interval; OR: odds ratio

^**a**^ ORs and 95% CIs for partial factors cannot be estimated, because the rate of these variables in the case or control was 0 or 100%

^**b**^ Hypertensive patients were defined as those who had a systolic blood pressure ≥ 140 mmHg and/or diastolic blood pressure ≥ 90 mmHg [21]

### Molecular typing of CRAB

**Fig. 4:**
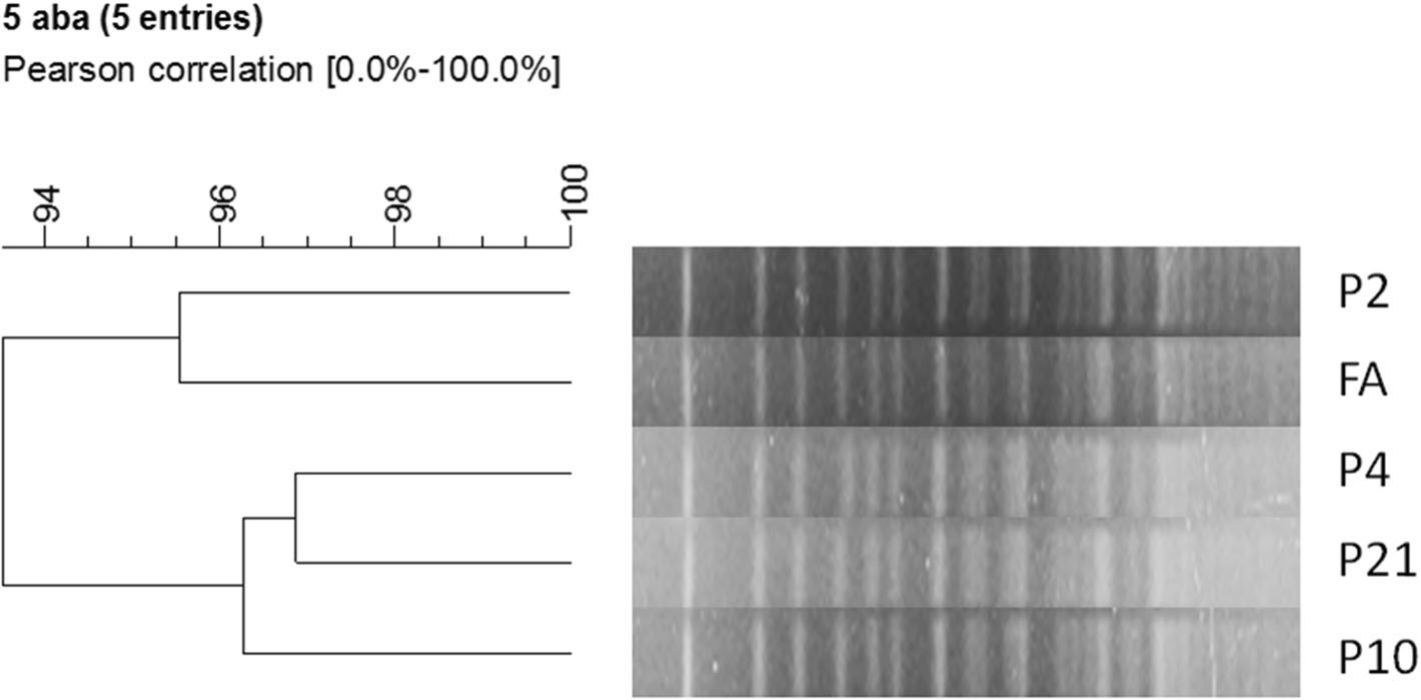
Gel map by CHEF-Mapper PFGE analysis of 5 CRAB isolates from clinical and environmental samples. P2,P4,P10,P21: bed number of inpatients; FA: faucet aerator

We analyzed molecular typing of 5 strains, including 1 strain isolated from the faucet aerator in the dining room, 1 strain isolated from the clinical samples of the first case that was infected before admission, and 3 strains isolated from the clinical samples of patients with HAI. Molecular typing results revealed that all 5 strains belonged to the same clone and this cloned strain caused the outbreak, as shown in Fig. 4.

### Hand hygiene compliance

**Fig. 5:**
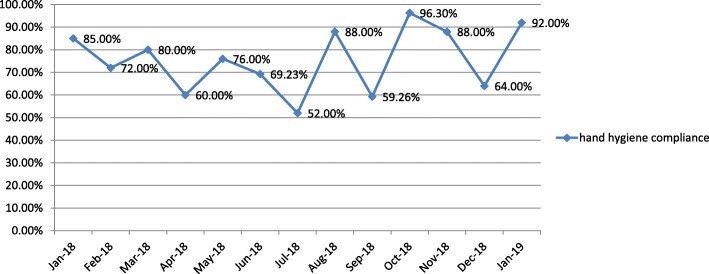
Hand hygiene compliance rate from January 2018 to January 2019 in NSICU. The hand hygiene compliance rate in December 2018 was at a low level throughout the year. Hand hygiene compliance monitoring happened to be done before the outbreak.

The hand hygiene monitoring team conducted a hand hygiene compliance observation on December 26, 2018 in the NSICU. Compared with the other months in 2018, the hand hygiene compliance rate in December was at a lower level, which was lower than the annual average of 74.15%, as shown in Fig. 5.

## Discussion

In the present study, environmental microbial sampling results showed that potable water and the inner surface of the proximal end of the water outlet tested negative for CRAB, and only one isolate from the faucet aerator was found to be culture positive. The molecular typing results showed that CRAB isolated from the faucet aerator and CRAB isolated from the clinical specimens had the same cloned strain. Previous studies have discussed the role of faucet aerators as a reservoir for bacteria [22, 23]. Weber and Kappstein concluded that low-level contamination of potable water led to contamination of the faucet aerators with subsequent bacterial amplification [24, 25]. But in Wang’s study, pathogens were thought to contaminate the faucet when it was used by the medical staff, rather than contaminating the water supply system [12]. It was difficult to determine whether the primary source of faucet contamination was municipal water, pipeline, or the hands of the medical staff. In our study, based on the analysis of sampling results and hand hygiene compliance results, we believed that contamination of the faucet aerators might have occoured from splashes originating from handwasing by the HCWs, instead of low-level contamination of potable water. It should be emphasized that because HCWs used the contaminated faucet to wash their hands, new cases of infection and colonization occurred on the 3rd and 4th day after the emergency response, with an interval of more than 48 h. Horcajada concluded that when a faucet is contaminated, contact precautions can fail, because HCWs wash their hands with contaminated water [26]. Successful control of the outbreak by prohibiting the use of aerators also supported our hypothesis that a contaminated aerator played the role of a key environmental reservoir during the outbreak..

In our NSICU, traditional mechanical foot-operated faucets are used instead of electronic sensor faucets. The aerators of these faucets are made of several wire meshes to filter impurities in water and to prevent splashing. However, these wires provide space for the propagation of pathogenic microorganisms. Walker’s research showed that the presence and type of aerator on the faucet was a factor involved in water contamination [27]. In high-risk areas, Kappstein recommend either the removal of aerators or use of aerators without wire meshes that do not collect sediments or lead to water stagnation, and regular cleaning of aerators [25]. In view of the use of the aerator and the structure of the aerator, which might cause aggravated pollution, we believed that the management of faucet aerators in high-risk areas, such as the NSICU, should be given greater attention.

The aggregation of CRAB detection time in these NSICU patients was obvious, but the spatial distribution of beds was more dispersed. The case-control study showed that risk factors, such as medical group, invasive operation, and underlying diseases, were not the suspected causes of the outbreak. The outbreak strain was found only in the dining room, which was a busy room with a greater amount of staff traffic than in other areas, and the faucet was used frequently for hand washing. We believed that the faucet aerator was contaminated by splashes originating from handwashing by the HCWs,and it might have become an environmental reservoir of CRAB during the outbreak. Subsequently, during the hand washing process, the CRAB-colonized faucet contaminated the hands of the HCWs, which became the carrier of CRAB and spread CRAB to the ICU inpatients. Patients in the NSICU often have coma or confusion, and they need to stay in bed for a long time. Compared with other ICU patients, there are more instances of tracheal opening and assisted suction, and the risk of HAI is greater; even low-level contamination can result in HAI [28, 29]. *A. baumannii* was the main challenge in HAI prevention and control in our NSICU. Its carbapenem-resistance rate reached 92.70%, and the isolation rate was 40.37% [30]. The bacterial reservoir (faucet aerator), the route of transmission (hands of the HCWs), and the susceptible population (NSICU inpatients) constitute the CRAB transmission chain in the outbreak.

We used a set of emergency response procedures developed by our own hospital to discover and successfully contain the outbreak in a timely manner. It was difficult to operate by simply using the “incidence above the previous level” mentioned in the guideline as a standard for the emergency response [14], because occasionally it was not easy to judge HAI cases, and doctors often needed to use the efficacy of antibiotics to assist in the judgment, but the timeliness of the emergency response could not withstand such long delays. In addition, our previous research showed that positive detection of *A. baumannii* in patients has a distinct seasonal distribution [31], which has been discussed in many previous studies [32–34]. The incidence of HAIs caused by *A. baumannii* differs with seasonal changes, and therefore, it is not appropriate to judge whether HAI outbreaks may occur according to the morbidity level. We believe that a sensitive emergency response initiation standard is the key to controlling HAI outbreaks. This standard may only be suitable for use in our own ICU, as it is based on our long-term experience in dealing with HAI outbreaks in our ICU (Additional file 1), which has its own unique architectural layout, bed layout, and infection control features. Although the suitability of this standard may be limited, our approach can provide other hospitals with a new way of strategizing to control HAI outbreaks.

### Study limitations

Because the intensive infection control measures were carried out before environmental microbial sampling, only one positive environmental specimen was detected. This led to failure to understand the situation when the NSICU was the most contaminated. In addition, we used only pre-moistened cotton swabs for sampling, and we did not use sampling sponges, whose better capture ability had been confirmed in previous studies [35, 36].

We did not sample the hands of the HCWs after they washed them under the contaminated faucet in the dining room, and we sampled only the hands of the medical staff that was undergoing medical treatment. Therefore, in the chain hypothesis of CRAB transmission, we could only infer from the results of hand hygiene compliance, and there was a lack of the most direct evidence.

We suggest that environmental sampling should be carried out as much as possible before the implementation of emergency measures in future CRAB outbreak investigations to obtain more abundant clues for pathogen transmission.

## Conclusion

This study highlights the importance of faucet aerators. In high-risk areas, we recommend that faucet aerators should not be used during an outbreak or they should be disassembled and cleaned.

## Supplementary information

**Additional file 1.** Background epidemiology of the NSICU and recent HAI outbreaks

## Data Availability

All data generated and analyzed during this study are included in this article.

## Abbreviations

CHINET: China Antimicrobial Surveillance Network
CRAB: Carbapenem-resistant *Acinetobacter baumannii*
GCS: Glasgow Coma Scale
HAI: Healthcare-associated infection
HCWs: Healthcare workers
NHSN: National Healthcare Safety Network
NSICU: Neurosurgical intensive care unit
PFGE: Pulsed-Field Gel Electrophoresis
RIT: Repeat Infection Timeframe
WHO: World Health Organization
