# Supplementary material for: Faucet aerators as a reservoir for Carbapenem-resistant Acinetobacter baumannii: a healthcare-associated infection outbreak in a neurosurgical intensive care unit
Source: Antimicrob Resist Infect Control. 2019 Dec 30;8:205. doi: 10.1186/s13756-019-0635-y (PMC6938019; doi:10.1186/s13756-019-0635-y)
Supplement: Supplementary file 1 — Additional file 1. Background epidemiology of the NSICU and recent HAI outbreaks [file 13756_2019_635_MOESM1_ESM.docx]

**Background epidemiology of the NSICU**

As shown in Figure 1, we analyzed the time distribution of hospital-acquired CRAB rates (Including infection and colonization, excluding specimen contamination and community infections) during the survey, in which January 2019 data began and ended from January 1 to January 19. We used the mean and 2times the standard deviation to determine the anomaly data during the outbreak. The results in Figure 1 suggested that the NSICU showed CRAB aggregation in January 2019.

*Hospital-acquired rates*

Mean of rate Mean **+** 2 times the standard deviation of the rate

Figure 1. The background epidemiology of the NSICU regarding CRAB

**Recent HAI outbreaks**

The following are two recent HAI outbreaks that occurred in our NSICU, one in 2016 and the other in 2017. In these two outbreaks, the positive detection of pathogens has a clear trend in both chronological order and bed distribution, which provides sufficient clues for the cause of the outbreak.

In 2016, China fully liberalized the two-child policy, resulting in a large number of NSICU nurses in pregnancy or childbirth. There were still enough nurses during the day, but in the evening shifts the nurses clearly showed a shortage. In our NSICU, the 20-22 beds used for isolation were handled by a nurse during the day, but at night, their responsible nurse also taken care of the 16-17 beds. In May 2016, a CRAB outbreak occurred in the NSICU. As shown in Figure 2, after the CRAB was taken out of the isolation room by the nurse, it gradually spread in the other two rooms. Then our NSICU applied to the nursing department to configure enough nurses.


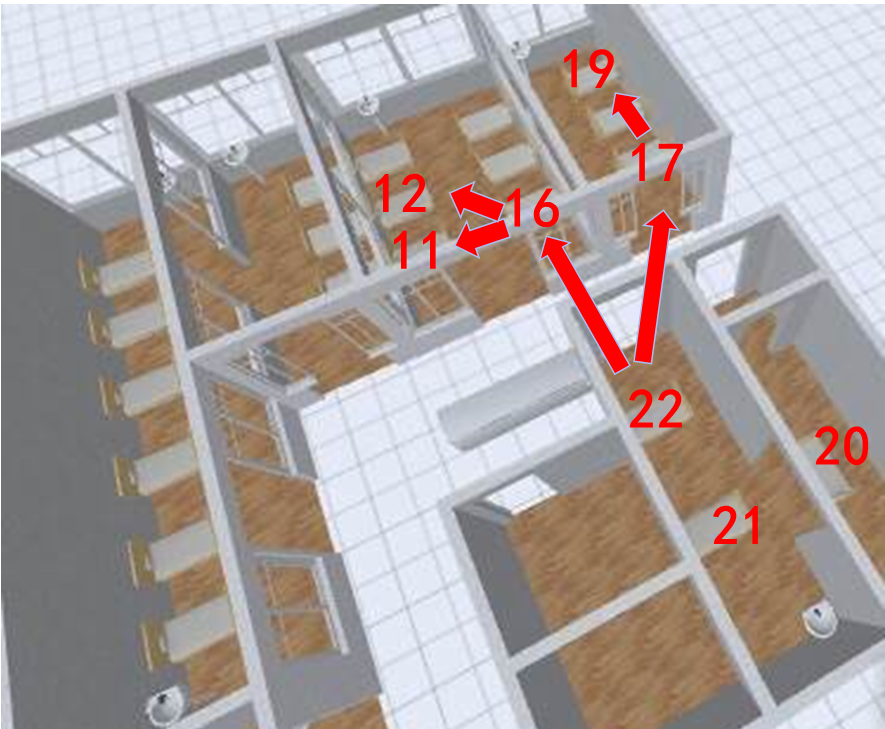


Figure 2. Schematic map of CRAB outbreak in 2016.

In April 2017, our NSICU issued a HAI outbreak due to CR-Pseudomonas aeruginosa. As shown in Figure 3, CRPA was first detected in clinical specimens of the 13-bed patient, followed by 12--bed, 11--bed, and 16-bed patients. In the subsequent environmental sampling, a CRPA consistent with the patient's clinical specimen was detected in the inner wall of the sink next to the 13-bed. The sink next to the 13-bed has a big problem, it is too close to the bed (just 1 meter), but the position of the sink cannot be changed because of the drainage pipe setting problem. Then our NSICU used a chlorine-containing disinfectant to immerse this sink once a day, but did not pay enough attention to the faucets or aerators.


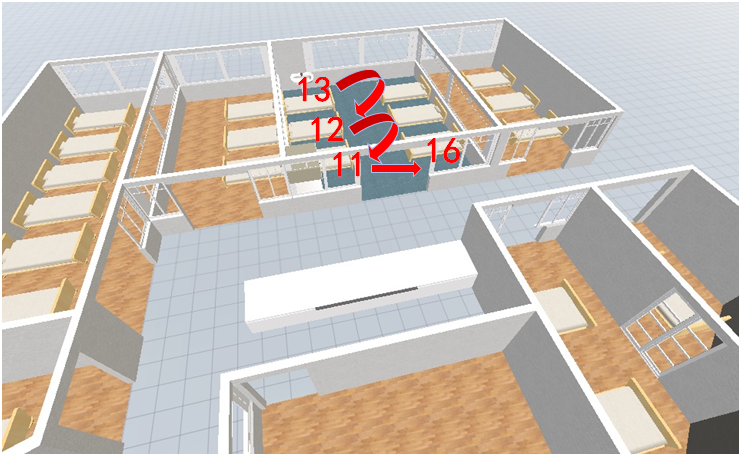


Figure 3. Schematic map of CRPA outbreak in 2017.
